# Openness policies and financial development in Ghana: An ARDL approach

**DOI:** 10.1016/j.heliyon.2024.e25074

**Published:** 2024-01-28

**Authors:** Mubarik Salifu, James Atta Peprah, Joshua Sebu, William Godfred Cantah

**Affiliations:** a Department of Economic Studies, School of Economics, University of Cape Coast, Cape Coast, Ghana; b Department of Economics, University for Development Studies, Tamale, Ghana; c Department of Applied Economics, School of Economics, University of Cape Coast, Cape Coast, Ghana; d Department of Data Science and Economic Policy, School of Economics, University of Cape Coast, Cape Coast, Ghana

**Keywords:** Financial development, Trade openness, Financial openness, ARDL, Ghana

## Abstract

Purpose: This study investigated the relationship between openness policies (trade and finance) and the degree of financial development in Ghana.

Design/methodology/approach: The data for this study were extracted from the World Bank's World Development Indicators from 1960 to 2020. The data consists of annual time series data for Ghana. This study employs the ARDL bounds testing approach for cointegration to estimate the short- and long-run effects of openness policies on financial development in Ghana. In addition, a Markov Switching Model is used as a robustness check to estimate the effect of openness policies and financial development and to handle issues of structural breaks in the dataset.

Findings: The study finds that openness policies have a substantial impact on financial development in Ghana and supports the McKinnon–Saw hypothesis. Specifically, financial and trade openness have positive effects on financial development in the long run. However, in the short run, trade openness is the only significant determinant of financial development. The results from the Markov model also indicate that the effect of openness policies on financial development is positive in high and low regimes of financial development, even though foreign investment does not affect financial development in less volatile environments.

Originality/value: This study examines the relationship between openness policies (trade and finance) and the degree of financial development in Ghana.

Research implications: This study suggests that government policies should focus on enhancing trade agreements to increase trade volumes and promote financial development in the long run. Additionally, reducing restrictions on capital movement across borders could encourage capital inflows and competition among financial institutions, leading to greater efficiency and development of the financial sector. Moreover, policies that promote financial openness can promote long-term financial development in the long run. Therefore, policymakers should work towards liberalizing capital accounts and promoting foreign investment. However, policymakers must be cautious about the potential short-term effects of such policies and work towards mitigating any negative impacts.

## 1 Introduction

Since the financial crisis of 2007–2008, policymakers and scholars have paid considerable attention to financial development in the context of economic recovery. Financial growth is a key contributor to economic progress and rising living standards. This is because increased financial development boosts financial market efficiency and available resources, the rate of technological advancement and capital accumulation, and the general importance of the financial system and investments [[1], [2], [3], [4]]. It has been demonstrated that financial development can lessen the financial constraints that businesses face, assist with the management of risk, increase monetary policy efficiency, and broaden the options available for fiscal policy, as well as possibly boost economic growth, as indicated in the works of [[5], [6], [7], [8]].

The interest group financial development theory proposed and developed by Rajan and Zingales [9](2003) asserts that the liberalization of an economy to trade with other economies is a significant driver of financial development, as an increase in the export of ideas and investments by domestic businesses typically increases the demand for external financing in the domestic economy. Openness policies are usually conceptualized to include trade and financial openness in an economy, such that the inflow and outflow of trade and finance into an economy will imply that there is increased openness within an economy. The degree of financial development in industrialized and developing nations has diverged significantly over the past few decades [10,11]. When measured using the ratio of private sector credit to GDP, it was found that the levels of financial development have been steadily increasing for developed countries, while the levels for developing countries have stagnated [12]. Thus, it is important to study the underlying causes of such disparities.

Researchers have identified that the openness of an economy to trade and financial market liberalization and development considerably impacts long-term financial development. Theoretically, financial openness suggests that liberalizing a financial system may affect financial market expansion through various channels. According to McKinnon and Shaw [13,14], liberalization of the financial system has the potential to reduce the extent of financial repression in protected markets by making it possible for real interest rates to approach the equilibrium of competitive markets. Second, the abolition of capital limitations enables both local and foreign investors to better diversify their portfolios. These two concepts may be summarized as follows: financial openness may reduce capital costs and improve borrowers' access. The third way in which financial openness may impact financial growth is through the removal of weak institutions, which often increases the efficiency of the financial system and generates demand for financial system improvements. To attain financial openness, barriers to the cross-border flow of money and financial services must be reduced. In addition, cross-border financial relations must be developed and expanded, and the unfavourable treatment of foreign capital and international investors must cease [15,16].

According to the literature, trade openness affects financial development. While some studies [[17], [18], [19], [20]] have developed a significant negative association between trade and financial development, others have established negligible and hence unclear conclusions on the relationship [[21], [22], [23]].

The following voids in the existing body of knowledge served as impetus for conducting this inquiry. There is a rapidly expanding body of literature on trade and financial growth. This body of literature can be found all over the internet. However, few studies have described how trade and financial openness contribute to financial development, particularly given the scant evidence on the contribution of financial development to economic growth and the efforts made by sub-Saharan African governments to ensure increased trade volumes and capital account liberalization. In addition, given McKinnon-Shaw's [13] conclusion that financial openness plays a major role in financial development, this study explores the long- and short-run effects of both trade openness and financial openness on financial development in Ghana.

### 1.1 Insights from related literature

The link between finance and trade openness and financial development has been the subject of many studies and has been shown by a few different studies. Arif and Rawat [17] observed that financial development is significantly affected by trade and openness. They suggest that while financial openness has a negative influence on financial development in South Asia, trade openness has a favourable impact; nevertheless, their simultaneous openness is not substantiated. This finding supports the hypothesis proposed by Rajan and Zingales [9] that trade and financial openness are fundamental for financial development. According to Rajan and Zingales, trade openness and capital flows can weaken the industrial opposition to financial development. This view contradicts McKinnon's [13] ideas, as cited in Refs. [24,51], who argued that financial liberalization should follow trade liberalization and that capital account opening should be the final step in the liberalization process. Memdani [25] concluded that, trade openness increases economic development, with a greater link for high-income countries and a lesser link for middle-income and low-income countries.

The degree to which international trade is liberalized is seen by some to affect the growth of both the supply and demand sides of the financial industry. According to Rajan and Zingales [9]as cited in the works of [1,17,50], trade openness is necessary to improve financial development because it boosts international competition in domestic markets. They explained that global competition hurts domestic producers' profitability and forces them to make more significant investments to compete with foreign products. Owing to the sourcing and utilization of these incumbents' cash, the financial industry will improve, opening up prospects for new companies to quickly raise capital for growth.

Although little research has been conducted on the Ghanaian economy, the literature on openness policies and financial growth is extensive. Using data from low-income nations, Baltagi et al. [26] discovered that the marginal effect of capital account liberalization on financial growth is relatively significant. They find that the expansion of the banking sector and financial and trade openness are positively connected in both developing and developed countries. Their findings contradict Rajan and Zingales' [9] claim that trade and financial openness are required as preconditions for financial growth for various political and economic reasons.

According to Mishkin [27], a certain level of financial development is necessary for financial development to react to the degree of financial openness. They claim that, to benefit from financial globalization, several requirements must be satisfied. These prerequisites include strengthening the legal system, developing strong corporate governance, eradicating corruption and establishing strong property rights. Trade openness increases the demand for new financial goods and services, reduces income volatility, and encourages financial growth [28]. According to Kurihara and Fukushima [29], this reduces domestic shock sensitivity and increases a nation's susceptibility to external shocks and international competition.

Research suggests that financial openness indirectly benefits GDP by affecting financial development, institutional development, and macroeconomic discipline. Higher financial development leads to greater manufactured exports and a favourable trade balance, especially in highly established financial systems. Capital account liberalization is linked to greater financial sector depth in advanced and developing economies [1,30].

Shahbaz et al. [31], discovered a short-run unidirectional causal relationship from financial development to trade openness for Indonesia, while Farhani and Ozturk [32] found a short-run Granger causality from financial development to trade openness for Tunisia. On the other hand, Gries et al. [33] found little to no evidence supporting a causal link between financial development and trade openness in 16 sub-Saharan African countries. Menyah et al. [34], found similar results for 21 Sub-Saharan African countries, with no evidence of causality between financial development and trade openness.

Considerable emphasis has been placed on empirical studies investigating the relationship between finance, trade openness, and financial development [17,26,35]. For instance, Herwartz and Walle [30](2014) revealed that, with certain exceptions in OECD and Latin American economies, high trade openness tends to reinforce the growth-promoting effect of financial development, whereas high financial openness often inhibits it. Gnangnon [24] found that increased trade openness helps developing countries meet the Sustainable Development Goals (SDGs) by 2030 by diversifying external financial flows for development, especially in the least developed nations. Bandura [35] found that trade and financial openness have weak positive impacts on financial development in sub-Saharan Africa, with institutions contributing positively to private credit by banks. Kim et al. [22], found that trade openness and financial development are complementary in the long run and substitute each other in the short-run for non-OECD countries. However, the impact of financial development on trade is found to be insignificant for OECD countries. The researchers discovered a non-linear relationship in the long run, with declining trade responses as financial development progressed.

Chortareas et al. [36] found that the long-run connection between finance and output only holds after considering economic openness. Developing countries appear to benefit more from trade openness, whereas financial openness is more crucial for advanced countries. Ibrahim and Sare [19] reveal that human capital and trade openness are equivalent in their ability to affect Africa's financial development, with trade openness having a stronger effect on private credit than on human capital.

Qamruzzaman and Jianguo [37] observed that using panel nonlinear autoregressive distributed lagged data from 1990 to 2017, except for developing countries, financial development, trade openness, and foreign capital flows show long-run asymmetric correlations with renewable energy consumption. Menyah et al. [34] concluded that the impact of trade liberalization and financial development on economic growth in African countries has been minimal, indicating that recent initiatives have not had a major impact on growth.

Abdallah [38] discovered that trade openness is a necessary condition for capital account liberalization, while banking sector development is a prerequisite for equity market development. Awili and Ahmed [39] found a positive interdependence between financial development and liberal trade policies. According to Jaud et al. [14], liberalization of the stock market in liberalizing countries expedites the removal of inefficient exports and enhances export performance in the long run, with international investors contributing uniquely to better resource allocation in the actual economy.

Rajan and Zingales [9] suggest a connection between commercial activity, financial openness, and financial advancement, but their findings may be insufficient or deceptive. Baltagi et al. [26] examined the impact of trade and financial openness on economic development, finding that stronger financial repression regulations in an economy open to commerce but restricted to capital account transactions would end the economic boom, making free trade and closed capital accounts unviable.

In conclusion, the effects of trade and financial openness on financial development are inconclusive. Overall, while openness seems to be positively linked to financial development, evidence for developing countries is less clear. Furthermore, the coverage of African countries in previous studies has been typically limited. In addition, there is less evidence in Ghana on the role of financial openness and trade openness in financial development. This study uses time-series data to explore the possible long-term consequences of increasing financial and trade openness on financial sector development.

## 2 Data and methods of analysis

### 2.1 Data

This study uses annual time-series data for Ghana. The data span from 1960 to 2020, despite missing observations for some variables. Data for the analysis were obtained from the World Bank's World Development Indicators. The ratio of domestic credit to the private sector as a percentage of GDP was used as a measure of financial development. Financial openness was measured using the ratio of net capital inflows to FDI to GDP. We used the GDP growth rate to calculate the impact of the economic growth rate on financial development. We also used trade openness to measure the effect of opening economies on external trade. This was calculated as the ratio of the sum of exports and imports to GDP. Inflation is measured using changes in the consumer price index.

### 2.2 Empirical estimations

Following previous work on the openness-growth nexus, the study adopts the following model

(1) $${FD}_{t}=f\left({financial\;openness}_{t},{trade\;openess}_{t}\right)$$

For the two openness measures, we set the following models:

(2) $${\text{FD}}_{t}=\gamma_{0}+\gamma_{1}{financialopenness}_{t}+\gamma_{2}{tradeopenness}_{t}+\gamma_{3}{\text{growth}}_{t}+\gamma_{4}{\text{investment}}_{t}+\gamma_{5}{\text{inflation}}_{t}+\gamma_{6}{\text{expenditure}}_{t}+\varepsilon_{t}$$

From equations (1) and (2), ${\text{FD}}_{t}$ measures the level of financial development. The ratio of domestic credit to the private sector as a percentage of GDP was used as a measure of financial development.

${financialopenness}_{it}$ is the financial level proxied by the percentage of capital inflows to GDP. Higher ratios are expected to result in higher levels of financial development. Computed as $\frac{net\;private\;capital\;inflows}{Gross\;Domestic\;Product}\times 100$. Other measures used in the literature include banks’ international activity ratios and FDI inflows to the GNP ratio. The ratio measures the degree of international linkage in the real sector, as expressed in foreign direct investments.

${tradeopenness}_{t}$ It measures the openness of the economy to external trade. This is also seen as a measure of trade liberalization. It sums up the total exports of an economy and its total imports and expresses that as a percentage of GDP. The more liberalized trade becomes, the higher the chances of developing the financial sector to finance the increased trade volumes.

${Investment}_{t}$ measures the level of private investment in the economy. This is proxied by Gross Fixed Capital Formation, computed as a percentage of Gross Domestic Product.

${inflation}_{t}$ measures annual changes in the prices of goods and services in the economy. It is measured using the percentage changes in the consumer price index. As Beck et al. [40], indicate, high inflation erodes the purchasing power of individuals, particularly those with lower income. This can make financial services and products, such as banking fees or loan interest rates, less affordable and therefore less accessible. This is expected to dampen the financial development.

${growth}_{t}$ is the annual change in the gross domestic product. The growth rate of an economy's GDP indicates its health. Therefore, it is expected that increasing GDP growth rates could generate sufficient economic activity and propel the financial sector to grow.

${expenditure}_{t}$ measures the extent of government financial expenditure on goods and services as a percentage of the total GDP. It is expected that government spending on social safety nets and welfare programs can mitigate economic shocks and reduce the financial vulnerability of the population. When individuals feel secure and trust the government's ability to manage the economy, it fosters a conducive environment for financial development.

$\varepsilon_{t}$ and t is the error and time variables, respectively.

### 2.3 Estimation techniques

This study employs the ARDL bounds testing approach for cointegration to estimate the short- and long-run effects of openness policies on financial development in Ghana. To implement the bounds testing procedure, the conditional ARDL error correction model (ECM) is modelled using Equation (2) as follows:

(3) $${\Delta \text{FD}}_{t}={\;\Omega }_{0}+\delta_{1}{\;\text{FD}}_{t-1}+\delta_{2}{\;\text{growth}}_{t-1}+\delta_{3}{\;\text{inflation}}_{t-1}+\delta_{4}{\;financialopeness}_{t-1}+\delta_{5}{\;tradeopeness}_{t-1}+\delta_{6}{\;\text{expenditure}}_{t-1}++\delta_{7}{\;\text{investment}}_{t-1}+\sum_{i=1}^{n}{\beta_{1}\Delta \text{FD}}_{t-1}+\sum_{i=0}^{n}{\beta_{2}\Delta \text{growth}}_{t-1}+\sum_{i=0}^{n}{\beta_{3}\Delta \text{inflation}}_{t-1}+\sum_{i=0}^{n}{\beta_{4}\Delta financialopeness}_{t-1}+\sum_{i=0}^{n}{\beta_{5}\Delta tradeopeness}_{t-1}+\sum_{i=1}^{n}{\beta_{6}\Delta \text{expenditure}}_{t-1}+\sum_{i=1}^{n}{\beta_{7}\Delta \text{investment}}_{t-1}+\mu_{t}$$

From equation (3), Δ is the first difference operator, and all other variables remain as already defined. An F-test is conducted to establish the joint significance of the coefficients. The F-test has a non-standard distribution and depends on whether the variables included in the ARDL model are I(0) or I(1), the number of regressors, and whether the ARDL model contains an intercept or trend.

The null hypothesis of no cointegration is tested using the joint significance test as follows:

$H_{0}:=\delta_{1}=\delta_{2}=\delta_{3}=\delta_{4}=\delta_{5}=\delta_{6}=\delta_{7}=0$ against the alternative hypothesis of

$$H_{1}:\delta_{1}\neq 0,or\delta_{2}\neq 0,or\delta_{3}\neq 0,or\delta_{4}\neq 0,or\delta_{5}\neq 0,or\delta_{6}\neq 0,or\delta_{7}\neq 0\text{.}$$

If the computed F-statistic is less than the lower bound critical value, we do not reject the null hypothesis of no cointegration. Alternatively, if the F-statistic is greater than the upper bound of the critical values, the null hypothesis of no cointegration is rejected. The inference is inconclusive if the F-statistic lies between the lower and upper bounds. Once cointegration is established, the long- and short-run estimations of the parameters are established.

Assuming a long-run relationship exists among the variables, we estimate a conditional ARDL based on equation (2) so that we get:

(4) $${\Delta \text{FD}}_{t}={\;\Omega }_{0}+\delta_{1}{\;\text{FD}}_{t-1}+\delta_{2}{\;\text{growth}}_{t-1}+\delta_{3}{\;\text{inflation}}_{t-1}+\delta_{4}{\;financialopeness}_{t-1}+\delta_{5}{\;tradeopeness}_{t-1}+\delta_{6}{\;\text{expenditure}}_{t-1}++\delta_{7}{\;\text{investment}}_{t-1}+Ф{\text{ECM}}_{t}+{\;\mu }_{t}$$

From equation (4), all variables are previously defined. The lag length in the ARDL model is selected based on an Akaike Information Criterion (AIC) with a maximum of 4 lags. The AIC considers both the goodness of fit of the model and the number of parameters used to achieve that fit. It penalizes models with more parameters to avoid overfitting, which occurs when a model fits the data too closely and may not generalize well to unseen data. By penalizing complex models, the AIC encourages simplicity and parsimony, while still prioritizing good model fit. This trade-off is crucial in selecting an optimal lag order that balances model accuracy and complexity [41,42].

Finally, the short-run dynamic elasticities were also computed by estimating an error correction (ECM) model associated with the long-run estimates such that;

(5) $${\Delta \ln\;FD}_{t}={\;\beta }_{0}+\sum_{i=1}^{n}{\beta_{1}\Delta \text{FD}}_{t-1}+\sum_{i=0}^{n}{\beta_{2}\Delta \text{growth}}_{t-1}+\sum_{i=0}^{n}{\beta_{3}\Delta \text{inflation}}_{t-1}+\sum_{i=0}^{n}{\beta_{4}\Delta financialopeness}_{t-1}+\sum_{i=0}^{n}{\beta_{5}\Delta tradeopeness}_{t-1}+\sum_{i=1}^{n}{\beta_{6}\Delta \text{expenditure}}_{t-1}+\sum_{i=1}^{n}{\beta_{7}\Delta \text{investment}}_{t-1}+{\psi \text{ECM}}_{t-1}+{\;\mu }_{t}$$

Where, β, ***ξ***, φ, φ, τ, ɳ, ϰ, and ϧ represents the short-run elasticities. Also, ψ measures the speed of adjustment while ECM_t-1_ is the error correction term lagged by one period from equation (5).

**Fig. 1:**
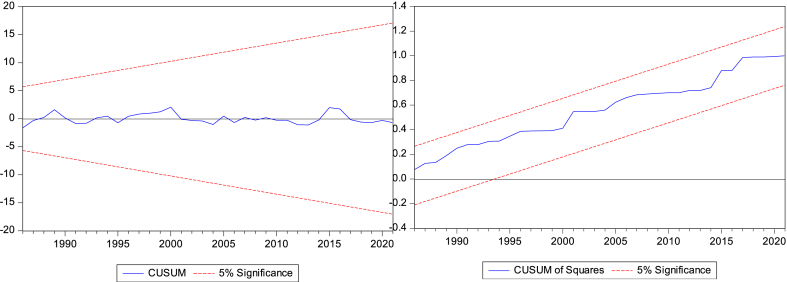
ARDL stability test

Using the Cumulative sum of squares and its square, we conduct a post-estimation test to verify the stability of the model. The model is deemed long-term stable if the values of CUSUM and CUSUM(SQ) are between the 5 % upper and lower boundaries (see Fig. 1 in Appendix A.

### 2.4 Test for linearity

To test for the presence of non-linearity, we use the model developed by Hansen [43]. It uses bootstrap replications to search for the presence of thresholds in the model such that our model is respecified as:

$${\text{FD}}_{t}=\gamma_{0}+\gamma_{1}{financialopenness}_{t}+\gamma_{2}{tradeopenness}_{t}+\gamma_{3}{\text{growth}}_{t}+\gamma_{4}{\text{Ginvestment}}_{t}+\gamma_{5}{\text{inflation}}_{t}+\gamma_{6}{\text{expenditure}}_{t}+\delta \left(tradeopenness\right)+\varepsilon_{t}$$

In our model, we use trade openness as the threshold switching variable for determining the non-linearity. The choice of the variable is because there exists a non-linear relationship between trade and financial development. Thus, testing of Null of No Threshold against the Alternative of Threshold as:

$$H_{o}:\delta =0$$

$$H_{1}:\delta \neq 0$$

## 3 Results and discussions

### 3.1 Unit root test

Using the ADF method for unit root, we test for the existence of unit root in the variables used in the study. The ARDL methodology requires that variables are cointegrated at either *I(0) or I(1).* The results in the table indicate all the variables are either stationary at I(0) or I(1). The result of the stationarity test is shown in Table 1.

**Table 1:** Unit root test using an Akaike information criterion.

Variables	Levels		First Difference		
	Intercept	Intercept and trend	Intercept	Intercept and trend	Level
FD	−1.491	−1.755	−8.592***	−8.517***	I (1)
Financialopenness	−1.602	−2.368	−7.653***	−7.589***	I (1)
expenditure	−3.276**	−3.669**	−5.853***	−5.777***	I (0)
inflation	−2.536	−6.386***	−12.957***	−12.857***	I (0)
growth	−5.021***	−5.599***	−5.892***	−5.838***	I (0)
tradeopenness	−1.213	−2.088	−2.550**	−2.451**	I (1)
investment	−2.124	−3.034	−2.075**	−1.944**	I (0)

According to the unit root results, all of the variables used in the study are stationary at either levels or first differences. This satisfies the conditions necessary for using the ARDL methodology for cointegration.

### 3.2 Correlation test

Table 2 shows the results of the correlation analysis of the variables used in the model. The correlation coefficients are not high enough to suspect the presence of multicollinearity among the variables used in the model.

**Table 2:** Pearson correlation test of variables used in the study.

Correlation Probability	FD	Financialopenes	growth	Inflation	investment	tradeopenness	expenditure
FD	1.000000						
	–						
Financialopeness	0.504995	1.000000					
	0.0000	–					
growth	0.388554	0.388915	1.000000				
	0.0020	0.0020	–				
Inflation	−0.505757	−0.395883	−0.353248	1.000000			
	0.0000	0.0016	0.0052	–			
investment	0.422540	0.393825	0.325183	−0.313870	1.000000		
	0.0000	0.0017	0.0106	0.0138	–		
tradeopenness	0.527917	0.512312	0.455385	−0.394224	0.506876	1.000000	
	0.0000	0.0000	0.0002	0.0017	0.0000	–	
expenditure	−0.236479	−0.086699	0.115792	0.203517	−0.265523	−0.015634	1.000000
	0.0665	0.5064	0.3742	0.1157	0.0386	0.9048	–

### 3.3 Test of non-linearity

There is no evidence of non-linearity or threshold given our sample evidence. From Table 3, we find that irrespective of the number of replications and trimming percentage, the results do not show evidence of threshold on the determinants of financial development conditioned openness as the regime-switching parameter. We therefore proceed to estimate a linear model.

**Table 3:** Test for non-linearity.

	Trimming percentage = 10 %				Trimming percentage = 15 %			
	500	1000	1500	2000	500	1000	1500	2000
Threshold Estimate	18.8146	18.8146	18.8146	18.8146	24.2438	24.2438	24.2438	24.2438
LM-test for no threshold	0.000	0.000	0.000	0.000	0.000	0.000	0.000	0.000
Bootstrap P-Value	0.978	0.899	0.994	0.786	0.967	0.874	0.789	0.882
Conclusion	No threshold	No threshold	No threshold	No threshold	No threshold	No threshold	No threshold	No threshold

### 3.4 ARDL bounds test for cointegration

We use the ARDL bounds testing approach to determine whether the dependent variable (FD) and the independent variable have a long-term cointegrating relationship. The null hypothesis that there is no cointegrating relation is rejected when the estimated F-statistics fall below the lower bounds. This is because the null hypothesis states that there is no cointegrating relationship.

Since the computed F-statistic of 6.263 is higher than the upper bound critical value of 5 % significance value as indicated in the results, we reject the null hypothesis and conclude that there exists a cointegration relationship between the level of financial development and the explanatory variables used in the model. We then proceed to estimate the long-run relationship between financial development and openness policies.

### 3.5 Estimated long-run effect of openness on financial development

Table 5 summarizes the results of the long-run relationship between openness policies and financial development. The results in Table 5 suggest that there exists a significant positive association between financial openness and financial development. The results indicate that a 1 % point increase in financial openness will lead to a 1.421 % point increase in financial development in the long run.

**Table 4:** Cointegration test using long-run bounds test.

F-Statistic	Level of Significance	Lower Bounds	Upper Bounds
6.263	10 %	1.99	2.94
	5 %	2.27	3.28
	1 %	2.88	3.99

**Table 5:** Long run results.

Dependent Variable: FD	
Variable	Coefficient
Financial openness	1.421*** (0.185)
expenditure	0.053 (0.097)
inflation	−0.042* (0.024)
growth	0.323* (0.172)
tradeopenness	0.108*** (0.032)
investment	−0.031 (0.122)
C	−2.323 (8.653)

One potential explanation for this relationship is that financial liberalization policies, such as capital account liberalization, may reduce financial repression in well-protected financial markets. This reduction in financial repression allows interest rates to rise to their competitive market equilibrium, promoting financial development. Moreover, the removal of capital controls facilitates better portfolio diversification for both domestic and foreign investors, leading to lower capital costs and improved access to capital for borrowers.

These findings are consistent with previous studies by Chin and Ito [44] and Hauner et al. [45], who have similarly demonstrated the positive impact of financial openness on financial development.

Overall, the findings of this study provide support for the notion that financial liberalization policies can play a crucial role in promoting financial development in the long run.

Additionally, trade openness influences financial development positively in the long run. The finding shows that a 1 % point increase in trade openness will result in a 0.108 % point increase in financial development in the long run. Trade openness can provide opportunities for developing economies to access international markets, attract foreign investment, and learn from more developed economies. Trade openness can also promote competition, which can incentivize domestic firms to improve their financial management and develop new financial products and services. The findings of this study are consistent with those found in previous studies, most notably the work of Bandura, [35]. According to Giday [46] the demand for external financial assets acts as an exogenous factor determining the level of a country's financial development. They contend that a country's comparative advantage in global commerce may impact production structure and external funding requirements. Consequently, a strong demand for external financing may be created when an economy specializes in items dependent on finances. This could therefore lead to the development of the financial sector.

The results also indicate a significant negative relationship between inflation and financial development. A 1 % point increase in the inflation rate will result in a 0.042 % point decrease in financial development. High and volatile inflation rates introduce uncertainty and increase risks in the economy. Uncertainty about future price levels makes it challenging for businesses and individuals to plan effectively, impacting investment decisions and long-term financial commitments. Financial institutions may face difficulties in accurately assessing the creditworthiness of borrowers, as inflation can erode the value of future repayment.

The growth rate of GDP in the long run is also a significant determinant of financial development. As indicated in Table 4, the relationship between the growth rate of GDP and financial development is positive and significant. A 1 % point increase in the GDP growth rate is expected to increase financial development by about 0.323 % points. As GDP grows, the demand for financial services increases. Financial institutions, such as banks, insurance companies, and investment firms, tend to expand their operations to meet the growing needs of businesses and individuals. The growth of the economy leads to higher incomes, increased investment opportunities, and greater financial transactions, all of which drive the expansion and diversification of financial institutions. This expansion enhances access to financial services and promotes the development of a robust and inclusive financial sector.

Government expenditure and investment expenditure are however not significant determinants of financial development in the long run in Ghana as indicated in Table 5. This could be because when the government spends more than it collects in taxes, it may need to borrow money or raise taxes to finance its deficit. This can have a negative effect on the private sector, which also relies on savings and loans for investment and consumption. The government's increased demand for funds can push up the interest rates, making borrowing more expensive and savings more attractive for the private sector. As a result, the private sector may reduce its spending and investment, which are vital for financial development. This phenomenon is known as the crowding out effect. This can therefore account for why government consumption expenditure does not affect financial development in the long run.

### 3.6 Estimated short-run relationship between openness and financial development

Table 6 indicates the results of the short-run relationship between financial development and openness policies. The ECM(-1) indicates how quickly long-run equilibrium is achieved when there are short-run shocks. It also indicates how the endogenous variables in the model respond to shocks in the exogenous variables in the model. A negative ECM(-1) reaffirms the cointegrating relationship earlier established. The results indicated that 51.1 % of the short-run shocks would be corrected in the following year.

**Table 6:** Short Run estimates of openness and financial development.

Variable	Coefficient
Δ financialopenness	0.214 (0.143)
Δ expenditure	0.209*** (0.047)
Δ expenditure (−1)	0.096* (0.052)
Δ inflation	−0.014** (0.006)
Δ inflation (−1)	−0.014* (0.007)
Δ growth	−0.078** (0.034)
Δ growth (−1)	0.178** (0.035)
Δ tradeopenness	0.028* (0.014)
Δ tradeopenness (−1)	−0.093*** (0.018)
Δ investment	0.048 (0.050)
Δ investment (−1)	0.198** (0.056)
CointEq(-1)	−0.511*** (0.067)
EC = FD - (1.4211*FINANCIALOPENESS -0.3226*GROWTH -0.0426*INFLATION -0.0318*INVESTMENT +0.1084*TRADEOPENNES + 0.0529*EXPENDITURE -2.3236)	

The short-run results indicate that financial openness does not have a significant impact on financial development. This may be a result of the fact that to participate effectively in global markets, financial openness often entails significant adjustments in the domestic financial system. These include adopting international financial standards, implementing regulatory reforms, improving infrastructure, and developing appropriate risk management practices. These adjustments are costly and time-consuming, and their effects on financial development may not be immediate. In the short run, the institutional and regulatory changes may still be incomplete, limiting the immediate benefits of financial openness for financial development.

Trade openness however is a significant determinant of financial development in the short run. The results in Table 6 indicate that in the short run, it is anticipated that a 1 % increase in trade openness will lead to a 0.093 % point decrease in financial development. One possible reason why trade openness may have a short-run negative effect on financial development is that trade openness may increase the exposure of the domestic economy to external shocks and volatility, which can undermine the stability and efficiency of the financial system. For example, trade openness may lead to sudden reversals of capital flows, exchange rate fluctuations, terms of trade shocks, and contagion effects from global crises. These shocks can impair the balance sheets of financial intermediaries, reduce the availability and quality of credit, and increase the risk of financial crises [15].

In the short run, the investment has a significant positive effect on Ghana's financial development. When investment in the short run increases by a percentage point, it is expected that the financial sector will respond with a 0.198 % point increase in its development. In the short run, investment can have an even more significant impact on financial development because it can boost confidence in the economy and attract foreign investment. When there is an increase in investment, it signals to investors that the economy is growing and has the potential for higher returns. This can lead to a positive feedback loop, where increased investment leads to more economic growth, which in turn attracts more investment. Empirical studies have also supported the positive relationship between investment and financial development in developing countries. For instance, Beck [7], found that investment has a positive and significant effect on financial development in a sample of 50 developing countries. Similarly, Levine [47] found that investment has a positive impact on financial intermediation in a sample of 80 countries.

Government expenditure in the short run also has a positive significant effect on financial development in Ghana. It is expected that a 1 % point increase in government consumption expenditure will increase financial development by a 0.096 % point increase in financial development. The results are consistent with the findings of Poku et al. [48], who also find that in the short run, financial development will respond positively to increases in government expenditure.

The results also show that the growth rate of GDP and the rate of inflation also significantly influence financial development in the short run. While the growth rate of GDP has a positive significant effect on financial development, the inflation rate has a negative significant effect on financial development. For the growth rate of GDP, a 1 % point increase will increase financial development by 0.178 % points. Also, a 1 % point increase in inflation will lead to financial development reducing by about 0.014 % points.

### 3.7 Diagnosis test

Various diagnostic tests were performed to ensure our model was free from various econometric errors, including normality, serial correlation, heteroskedasticity, and functional forms. The results are indicated in Table 7.

**Table 7:** Diagnostics test.

Functionality	Ramsey RESET Test F-statistic = 0.498 P-value = 0.485
Serial Correlation	Breusch-Godfrey LM Test F-statistic = 0.299 Prob. F (2, 34) 0.743
Heteroscedasticity	Breusch-Pagan-Godfrey Test F-statistic = 0.633 Prob-value = 0.866
Stability Condition	CUSUM = Stable CUSUMSQ = Stable

Using the Ramsey RESET test we tested for the specification errors in our model. The results of the test with an F-Statistic of 0.498 indicate that the model is correctly specified and does not suffer from any specification errors. The result of the serial correlation was tested using the Breusch-Godfrey LM Test. With an F-Statistic of 0.299, we can conclude that the model does not suffer from serial correlation in the error term. The results of the Breusch-Pagan-Godfrey Test of constant variance indicate that our model does not suffer from the problem of heteroskedasticity. With an F-statistic we conclude that our residuals are homoscedastic.

### 3.8 Variance inflation factor

Table 8 presents the results of multicollinearity using the variance inflation factor. The rule of thumb is that centred VIF values should be greater than 10 for multicollinearity to be severe. The results however indicate that multicollinearity is not present in the model.

**Table 8:** Multicollinearity test using the Variance Inflation Factor.

Variable	Centred
	VIF
FD(-1)	9.389345
Financialopennesss	9.599557
financialopenness(-1)	9.667342
growth	2.002176
growth(-1)	2.162355
growth(-2)	2.336063
Inflation	2.098908
Inflation(-1)	2.127408
Inflation(-2)	1.945323
Inflation(-3)	1.887183
Investment	9.172924
Investment(-1)	9.810981
Investment(-2)	7.044110
Tradeopenness	8.930561
Tradeopenness(-1)	9.259161
Tradeopenness(-2)	7.112691
Expenditure	5.433482
Expenditure(-1)	6.449227
Expenditure(-2)	6.651481
Expenditure(-3)	3.891325
Expenditure(-4)	3.304851
C	NA

### 3.9 Robustness checks

A Markov Switching model is further used to estimate the effect of openness policies and financial development. Due to the data-generating process of financial development and openness policies, various breakpoints could exist in the data points. To enable switching between regimes, the Markov switching is employed which can handle a lot of structural breaks [49]. The models are estimated with two regime-switching variables each. The regime-switching variables include the mean values, variances and variables of openness policies. Low regimes of financial development correspond to μ0 while high regimes correspond to μ1. The results of the Markov switching model are indicated in Table 9.

**Table 9:** Results of Markov switching model.

Variables	Coefficients	Standard Error
Mean (μ0)	1.147***	0.393
Mean (μ1)	3.721***	0.819
Variance (σ0)	−0.344*	0.179
Variance (σ1)	0.463***	0.131
Δforeign 0	−0.032	0.201
Δforeign 1	0.759***	0.118
Δtrade 0	0.071***	0.010
Δtrade 1	0.075***	0.013
Duration 0	14.63	
Duration 1	23.66	

The results in Table 9 indicate that in a low regime of financial development with low volatility, foreign investment has no significant effects on financial development. However, in the same regime, trade openness has a significant positive effect on financial development. A percentage point increase in trade openness will result in an increase in financial development by about 0.071 %. Thus, when financial development is less volatile trade openness increases financial development.

In a regime of high financial development, foreign investment and trade openness both significantly affect financial development. A percentage-point increase in foreign investment is expected to increase the level of financial development by about 0.759 % while a percentage-point increase in trade openness is expected to increase the level of financial development by about 0.075 %. The persistence of financial development in the various regimes tends to indicate that financial development is very persistent in high-growth regimes with higher volatilities than low low-growth regimes. The results show that it could take about 23.66 years to transition from a high financial development regime to a low regime when there is a shock to the economy.

### 3.10 Transition probabilities

Transition probabilities describe the likelihood of moving from one state (0) or regime to another (1) at each time step. The rows of the matrix represent the current state, and the columns represent the next state. The results of the transition probabilities are presented in Table 10.

**Table 10:** Results of the transition probabilities.

Regime	1	2
1	0.9317	0.0683
2	0.0423	0.9577

The results indicate that the model tends to be persistent in its current state when there is low growth and low volatility. The model is 93.17 % more likely to stay in a low growth, low volatility regime with a duration of about 14.63 years while in low growth, high volatility regimes, the model is 6.83 % more likely to transition to a high growth, high volatility regime. However, we see that the model is more persistent in the high growth, high volatility regime with a probability of 95.77 of staying in that regime. The model could persist in this state for about 23.66 years.

## 4 Conclusions and recommendations

The relationship between capital restrictions, financial system integration, and economic growth has been a topic of discussion for many years. As economies become more interconnected, the financial sector's importance as a facilitator of economic growth has increased. The theory of financial growth proposed by McKinnon and Shaw in 1973 suggests that financial development can be promoted by liberalizing capital accounts. This theory has been influential in shaping policies around the world, particularly in developing countries.

This study examined the relationship between financial openness, trade openness, and financial development. The study utilized the ARDL long run and bounds test estimation approach, which is a widely used method for examining the relationship between variables. The study controlled for other macroeconomic variables, such as inflation, economic growth, government spending, and investments. Data for the period 1960 to 2020 was used for the study. A Markov switching regression is also used to study how financial development responds to openness policies in different regimes.

The results of the study showed that financial openness and trade openness both have a positive impact on financial development in the long run. Financial openness had an insignificant effect on financial development in the short run, while trade openness had a significant negative effect. These findings suggest that policymakers should be cautious about the short-term effects of trade policies on the financial sector. While trade policies may be beneficial in the long run, their short-term effects may be harmful to the financial sector. The results also indicate that in the long run, government expenditure has an insignificant effect on financial development while in the short run government expenditure has a significant positive effect on financial development. Inflation has a significant negative effect on financial development both in the long run and short run. The growth rate also has a positive significant effect on financial development both in the long run and short run. The results from the Markov model also indicate that the effect of openness policies on financial development is positive in high and low regimes of financial development, even though foreign investment does not affect financial development in less volatile environments.

The study's findings support the idea that openness policies are beneficial for promoting financial development in the long run. However, policymakers must carefully consider the potential short-term effects of such policies on the financial sector. For example, policies that encourage foreign investment may lead to short-term fluctuations in exchange rates, which can have a significant impact on financial development. Policymakers must carefully balance the long-term benefits of openness policies with their short-term costs.

### 4.1 Recommendations

The literature on financial development documents a positive benefit of financial development to economic growth and as such special attention should paid to the development of the financial system of every economy. Given the stage of development of the Ghanaian economy, policies which promote trade, greater financial integration and removal of capital restrictions should be prioritized.

It is thus recommended that government policies should be targeted towards enhancing trade agreements with other countries such that it increases trade volumes, which could in turn enhance financial development in the long run. There should also be fewer restrictions on capital movement across borders to enhance an inflow of capital into the economy, increasing competition among financial institutions ensuring efficiency and consequently the development of the financial sector.

Policies that promote financial openness can be beneficial for promoting financial development in the long run. Therefore, policymakers should work towards liberalizing capital accounts and promoting foreign investment. However, policymakers must be cautious about the potential short-term effects of such policies and work towards mitigating any negative impacts. While trade policies may be beneficial for economic growth in the long run, their short-term effects may be harmful to the financial sector. Policymakers should balance the benefits and costs of trade policies to ensure that they do not have a significant negative impact on financial development in the short run.

## Disclosure statement

We, the authors of this research, declare that we have no competing interests.

## Funding

This study received no funding.

## Data availability statement

The data is available upon request.

## CRediT authorship contribution statement

**Mubarik Salifu:** Writing – review & editing, Writing – original draft, Validation, Methodology, Formal analysis, Data curation, Conceptualization. **James Atta Peprah:** Writing – review & editing, Writing – original draft, Validation, Supervision, Software, Conceptualization. **Joshua Sebu:** Writing – review & editing, Writing – original draft, Methodology, Formal analysis, Data curation, Conceptualization. **William Godfred Cantah:** Writing – review & editing, Writing – original draft, Validation, Software, Methodology, Formal analysis, Data curation, Conceptualization.

## Declaration of competing interest

The authors declare that they have no known competing financial interests or personal relationships that could have appeared to influence the work reported in this paper.
